# Corrigendum: Methylation Statuses of H19DMR and KvDMR at WT2 in Wilms Tumors in Taiwan

**DOI:** 10.3389/pore.2022.1610677

**Published:** 2022-08-08

**Authors:** Meng-Yao Lu, Wen-Chung Wang, Tai-Cheng Hou, Chen-Yun Kuo, Yen-Chein Lai

**Affiliations:** ^1^ Department of Pediatrics, National Taiwan University Hospital, Taipei, Taiwan; ^2^ Department of Obstetrics and Gynecology, Jen-Ai Hospital, Taichung, Taiwan; ^3^ Department of Pathology, Jen-Ai Hospital, Taichung, Taiwan; ^4^ Department of Medical Laboratory and Biotechnology, Chung Shan Medical University, Taichung, Taiwan; ^5^ Clinical Laboratory, Chung Shan Medical University Hospital, Taichung, Taiwan

**Keywords:** methylation, multiplex ligation-dependent probe amplification, Nephroblastoma, paternal uniparental disomy, Wilms tumor

In the published article, there was an error in the Funding statement. The funding statement for the grant number from Chung Shan Medical University was displayed as “CSMU-INT-108-2.” The correct statement is “This work was supported by a grant from Chung Shan Medical University (grant number CSMU-INT-108-12).”

The authors apologize for this error and state that this does not change the scientific conclusions of the article in any way.

